# An LSPR Sensor Integrated with VCSEL and Microfluidic Chip

**DOI:** 10.3390/nano12152607

**Published:** 2022-07-29

**Authors:** Fang Cao, Xupeng Zhao, Xiaoqing Lv, Liangchen Hu, Wenhui Jiang, Feng Yang, Li Chi, Pengying Chang, Chen Xu, Yiyang Xie

**Affiliations:** 1 Key Laboratory of Optoelectronics Technology, Ministry of Education, Beijing University of Technology, Beijing 100124, China; caofang950318@163.com (F.C.); zhaoxp2311@163.com (X.Z.); huliangchen163@163.com (L.H.); jiangwenhui1997@163.com (W.J.); yangfengqs@bjut.edu.cn (F.Y.); pychang@bjut.edu.cn (P.C.); xuchen58@bjut.edu.cn (C.X.); 2 State Key Laboratory of Integrated Optoelectronics, Institute of Semiconductor, Chinese Academy of Sciences, Beijing 100083, China; 3 School of Traditional Chinese Medicine, Capital Medical University, Beijing 100069, China

**Keywords:** sensor, localized surface plasmon resonance (LSPR), VCSEL, microfluidic, anodic aluminum oxide film

## Abstract

The work introduces a localized surface plasmon resonance (LSPR) sensor chip integrated with vertical-cavity surface-emitting lasers (VCSELs). Using VCSEL as the light source, the hexagonal gold nanoparticle array was integrated with anodic aluminum oxide (AAO) as the mask on the light-emitting end face. The sensitivity sensing test of the refractive index solution was realized, combined with microfluidic technology. At the same time, the finite-difference time- domain (FDTD) algorithm was applied to model and simulate the gold nanostructures. The experimental results showed that the output power of the sensor was related to the refractive index of the sucrose solution. The maximum sensitivity of the sensor was 1.65 × 10^6^ nW/RIU, which gives it great application potential in the field of biomolecular detection.

## 1. Introduction

Biosensors are usually devices for detecting and monitoring chemical substances and biomolecules, divided into many types according to different sensing methods [1]. The optical biosensor is an integrated real-time, fast, and portable sensor [2,3,4]. The optical biosensor detects the changes caused by the interaction between biomolecules through the optical readout system and converts the relevant information into exportable information for measurement [5,6]. The most popular optical biosensors use the localized surface plasmon resonance (LSPR) or surface plasmon resonance (SPR) effect of precious metals as the sensing principle. The SPR effect has been applied in the sensing field for nearly 40 years [7], and various configurations based on the prism and optical fiber have also been designed [8,9,10]. However, the sensors based on a prism structure have the problems of large volume and low integration [11]. Although the optical fiber sensors have a small volume and simple design, most optical fiber sensors have difficulty extracting and detecting samples simultaneously due to their structural characteristics. Therefore, the above two types of sensors cannot sufficiently realize the synchronization of integration and detection. Because LSPR can be directly coupled with photons and is easy to excite, the device for exciting LSPR does not need a prism device as complex as SPR [12].

In recent years, sensors based on the LSPR effect have been widely used in biosensors, medical diagnosis, catalysis, food detection, and other fields [13,14]. Especially since 2019, the widespread COVID-19 pandemic has brought severe illness and death for human beings. Therefore, it is essential to detect and prevent COVID-19 infection in time. Based on this, our research on biosensors has vital practical significance [15,16]. As we all know, the resonance peak of LSPR and sensor sensitivity are closely related to the material, size, and shape of the noble metal nanoparticles, and the refractive index of the surrounding media [17,18,19,20]. At the same time, the miniaturization and high integration of chips have become the development trend of the biochemical sensor chip of the future. Lu et al. proposed an LSPR sensor with high uniformity and high-density single-layer gold nanoparticles as the sensing layer, improving the sensitivity and reducing the detection limit through the proposed self-assembled template technology [21]. Kamyar Behrouzi et al. demonstrated an application of LSPR sensors based on gold nanoparticles to detect the SARS-CoV-2 nucleocapsid protein (n) in the visible range, in 2021. When gold nanoparticles bind to SARS-CoV-2 antigen-specific antibodies, the spectral color of the solution will change. However, it cannot detect very high protein concentrations [15]. Although these sensing platforms have realized the detection of biological substances, most of them have very complex manufacturing processes. Su Wei et al. proposed a low-cost refractive index sensor based on DVD-ROM, which adopts angle modulation and wavelength modulation, and the sensitivity is 520 nm/RIU [22]. Chen et al. proposed fixing gold nanoparticles on the inner wall of a microcapillary to realize LSPR sensing, which can recognize a large dynamic detection range with high adjustable sensitivity [23]. Fan et al. demonstrated a smartphone biosensor system based on the principle of LSPR and an integrated multi-channel microfluidic chip, which can be used to detect a variety of biological samples [24]. However, most of the existing optical sensors have low integration. The system depends on the external light source, which significantly improves the equipment cost and the complexity of the optical path. In 2020, Xie Y.Y. et al. reported a method of integrating a vertical-cavity surface-emitting laser (VCSEL) with the hypersurface and successfully realized the efficient shaping of the laser beam [25]. This research also adopts the method of VCSEL on-chip integration. On the basis of the research reported by Zhao et al., the preparation process of the sensing device was improved [26]. Using VCSEL as the sensing light source, the high integration of excitation and sensing, and the miniaturization of the sensing chip were realized, which increased the sensitivity of the device by an order of magnitude.

A new refractive index LSPR sensor integrated with VCSEL is proposed in this work. A VCSEL of a wavelength of 850 nm is used as the sensor’s light source, and a gold nanoparticle array and a microfluidic channel are integrated on the light-emitting end face. The gold nanoparticle array is used as the sensing layer, and the microfluidic channel is used as the injection chamber of the sensing process. By measuring the output optical power and spectral characteristics of VCSEL, we can systematically study the sensing performance of the new sensor. Metal nanoparticles with LSPR properties are usually prepared by focused ion and electron beam exposure. However, this process is often costly, inefficient, and cannot be prepared on a large scale. This study used anodic aluminum oxide (AAO) films to prepare gold nanoparticle arrays. The preparation process is simple, efficient, and available for large-area sensors. Compared with the reported sensors, our proposed device solves the problem of light source integration, and provides a new method for the integration and measurement of optical sensors. However, due to the influence of process factors, the initial optical power of different VCSEL units in the same chip is different, so the test process needs to find units with similar performance for comparison. At the same time, the experimental results are verified by the finite-difference time-domain method (FDTD), and the results are in good agreement with the experimental results.

## 2. Materials and Methods

### 2.1. Sensor Fabrication

**Figure 1:**
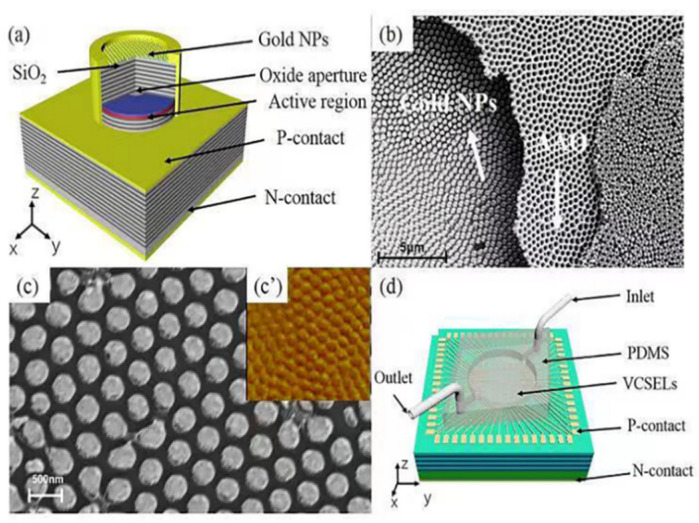
(**a**) Schematic diagram of VCSEL integrated with gold particles. (**b**) SEM of gold particles with a part of AAO film. (**c**) SEM diagrams of gold particles. (**c’**) AFM diagrams of gold particles. (**d**) Schematic diagram of the refractive index sensor chip with a microfluidic channel.

This work used a VCSEL with a laser band of 850 nm as the sensing light source. Figure 1a is a three-dimensional schematic diagram of the VCSEL integrated with a gold nanoparticle array, which shows the chip’s main structure and morphology integrated with a hexagonal gold nanoparticle array, taking a single laser as an example.

Anodic aluminum oxide (AAO) film was used as a mask in this experiment. An array of gold nanoparticles as a sensing layer was prepared on the surface of the VCSEL by magnetron sputtering. The preparation process was as follows: firstly, a VCSEL chip with a wavelength of 850 nm was prepared. Then, a layer of 500 nm SiO_2_ was passivated on the prepared VCSEL surface by PECVD to insulate the VCSEL from the gold nanoparticle array. Secondly, a layer of 240 nm PMMA adhesive was spin-coated onto the SiO_2_ passivation layer as the adhesive layer and dried at 120 °C on the drying table for 10 min. The third step was to transfer the AAO film. The AAO film was soaked in acetone solution for 3–5 min to remove the PMMA support layer and some impurities of the AAO film to improve the film quality. The treated AAO film was transferred to the surface of deionized water with a hydrophilic treated silicon wafer, and then transferred and bonded to the VCSEL surface to cover the laser table surface. To make the AAO film fit more closely with the substrate, it was necessary to heat it on a hot plate with a temperature of 150 °C for 10 s after transfer. The AAO film used in this study was hexagonal close-packed periodic film. The pore diameter D_AAO_ = 280 nm, the center distance of circular holes AAO = 450 nm, and the film thickness h_AAO_ = 500 nm. Then, reactive ion beam etching (RIE) was used to etch PMMA films. The patterned PMMA can improve the periodicity of AAO films. The magnetron sputtering of 10 nm Ti and 50 nm Au was performed on the end surface of the devices. Finally, the AAO was stripped off to obtain a periodic gold nanoparticle array, as shown in Figure 1b. The stripping process involved heating and boiling the chip with acetone and ethanol, respectively, and then drying it with a nitrogen gun. In a different way from the chips prepared by Zhao et al., the preparation process of gold nanoparticles was optimized by adjusting RIE etching conditions and sputtering rate of gold nanostructures [26]. It can be seen from the SEM that the gold nanoparticles prepared in this work had higher quality and better periodicity, as shown in Figure 1c. In addition, the quality of the gold nanoparticle array was improved by the annealing process.

After preparing gold nanoparticles, a PDMS microfluidic channel with an injection chamber needed to be bonded onto it, as shown in Figure 1d. After bonding the microfluidic channel, the complete refractive index sensor chip is shown which defines the inlet and outlet of the injection. The circular cavity of the microfluidic channel should cover the laser mesa area on the chip. For more details, the PDMS prepolymer and curing agent were evenly mixed at the ratio of 10:1, and the mixture was left to be bubble-free. Then, the PDMS prepolymer was poured into the master mold with pattern and dried at 80 °C for half an hour. PDMS is an ideal material for microchips with good light transmission and thermal stability [27]. To ensure the quality of light transmission, no residual bubbles were allowed to remain in the PDMS.

### 2.2. Simulation of Gold Nano Hexagonal Arrays

The finite-difference time-domain (FDTD) algorithm was applied to simulate its transmittance for the proposed hexagonal gold nanoparticle array. The software based on the FDTD method was developed by Canadian Lumerical company (Vancouver, BC, Canada). It can be used to study plasmas and nanooptics by solving Maxwell equations. By simulating the optical properties and electrical field distribution of nanostructures, we can design high-performance nanostructures to match the requirements of devices. Using 3D numerical simulation, the cylinder replaced the hexagonal gold nanoparticles, simulated in one cycle. The X and Y directions of the simulation area were set as periodic boundary conditions. The Z direction was set as a perfect matching layer (PML).

### 2.3. Characterization of the Gold Nanoparticle Array

After preparing the gold nanoparticle array, its morphology was characterized by scanning electron microscope (SEM-GeminiSEM 300; Shanghai, China). It was characterized by SEM with an accelerating voltage of 5 keV.

### 2.4. Result Test Method

After the device was prepared, we used the power (P)—current (I)—voltage (V) curve test system to detect the output tube power of the sensor chip. The test platform comprised a microfluidic pump (LSP01-1A; Shanghai, China) system, a microscope, a three-way rotating shaft, a probe table, a DC source (Keithley 236 DC; Cleveland, OH, USA), an optical power meter (PM400; Shanghai, China), a spectrometer (Agilent 86141B; Shenzhen, China), an optical power probe (S120C; Shanghai, China), an optical power semiconductor laser parameter analysis tester (Agilent 4156 C; Beijing, China), etc. The test platform injected current into the sensor chip to cause laser lasing, and the optical power probe could detect the change of its output optical power.

## 3. Results and Discussion

### 3.1. Comparison of Experimental and Simulation Results of Transmittance of Gold Nanoparticles

**Figure 2:**
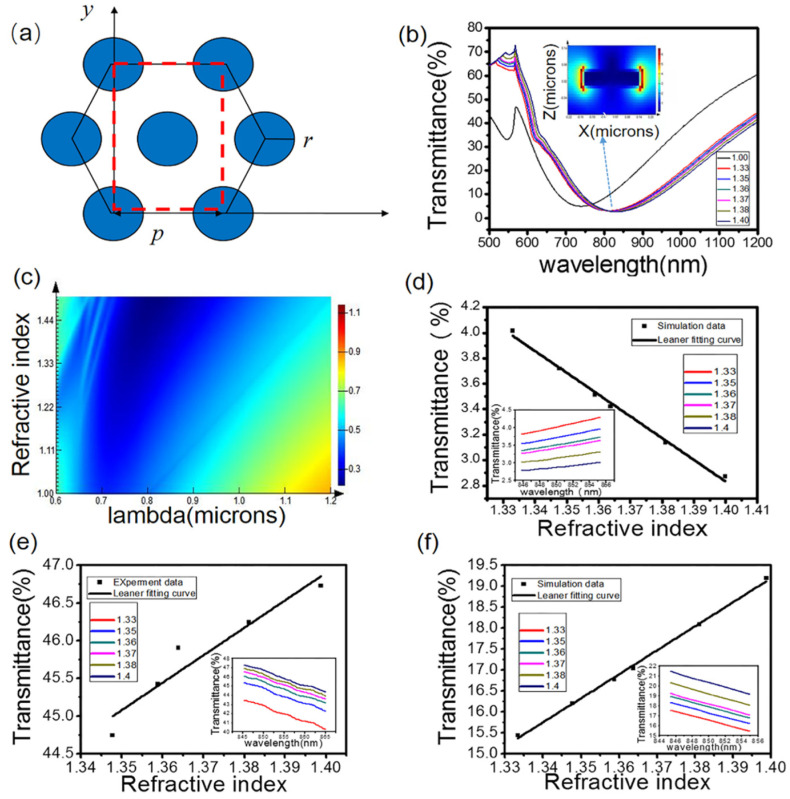
(**a**) Schematic diagram of a hexagonal array structure of single-crystal cell cycle. (**b**) Electric field distribution of cavity mode. (**c**) Variation of refractive index with a field strength of wavelength. (**d**) For the designed gold nanoparticle size, FDTD transmittance simulation sensitivity fitting results. (**e**) Test results of quartz glass transmittance. (**f**) For the actual gold nanoparticle size, FDTD transmittance simulation, and sensitivity fitting results.

We studied the transmittance properties of Au nanoparticle arrays prepared by AAO thin films. Figure 2a shows a schematic diagram of a hexagonal array structure with a single-cell cycle. The simulation model was established. The parameters of the gold nanostructure were period T = 450 nm, diameter R = 280 nm, height h = 50 nm, the height of Ti h = 10 nm, and the other parameters were the same as Au. An incident plane wave light source followed the positive direction of the z-axis, and a monitor was set to detect the transmission of the gold nanoparticle array. The transmittance spectrum of the gold nanoparticle structure was obtained, and the transmittance sensitivity of the device at the wavelength of 850 nm was fitted, as shown in Figure 2d. Although the simulated LSPR formant was not at 850 nm, the transmittance spectrum would move with the change in the environmental refractive index. The 850 nm wavelength was the band closest to the formant; therefore, we chose the VCSEL in this band for sensing. Figure 2b shows the electric field distribution of gold nanoparticles. It can be seen from the figure that the electric field around the gold particles at the light source wavelength of 850 nm was enhanced, and the spectrum also redshifted with the increase in the refractive index of the external solution, so the LSPR effect occurred and could be used for sensing. Figure 2c shows the change of background refractive index with the field strength of wavelength.

Gold nanoparticle arrays were integrated on quartz glass substrates, and bonded microfluidic channels. Then, the sucrose solution with refractive index (RI) of (1.333–1.3998) was introduced into the microfluidic channel at a steady flow rate of 1 mL/h from low to high, and its transmission spectrum was measured. Air needed to be introduced between sucrose solutions with different refractive indexes to realize the separation of solution samples. The effective volume of the sensing area in the channel was 3 μL. Therefore, the sample usage of this device of about 10 μL was enough. Figure 2e shows the transmission spectrum of the hexagonal gold nanoparticles array relative to RI measured experimentally. The figure shows the fitting curve of transmittance of the gold nanostructures in the 850 nm laser wavelength region; we could clearly observe that transmittance increased approximately linearly with the refractive index of the solution.

Comparing Figure 2d,e, it can be seen that there was some deviation between the experimental results. The reason may be that the periodicity of AAO film itself was not good, and the preparation process was limited, resulting in poor periodicity and deviation of particle size. We observed the gold nanoparticles prepared by AAO film under a scanning electron microscope, as shown in Figure 1c, which shows the SEM of gold nanoparticles. According to Figure 1c, the diameter of the prepared gold particles was about 400 nm, and the period could be considered 600 nm. Since the formants of gold nanoparticles with different sizes were located at different wavelengths, and the formant wavelength in Figure 2d was less than 850 nm, the transmittance at 850 nm wavelength decreased with the increase in the environmental refractive index. As shown in Figure 2f, the formant wavelength of the actually prepared gold nanoparticle structure was greater than 850 nm, so the transmittance at 850 nm wavelength increased with the increase in the environmental refractive index. Hence, the behavior of the two is opposite.

Figure 2f shows the transmittance results obtained by FDTD simulation of the size of the actually prepared gold nanoparticles and the fitting sensitivity at 850 nm. The figure shows that with the fitting curve of transmittance of the gold nanostructures in the 850 nm laser wavelength region, it can be clearly observed that the transmittance increased approximately linearly with the refractive index of the solution, within the studied range. In general, it matched the experimental results well, and the experimental and simulation results showed that the transmittance spectrum red-shifts when the ambient refractive index increases. Figure S2 of the Supplementary Materials is cited here.

### 3.2. Characterization of Sensing Performance

Using the new method, the size of the refractive index sensor can be smaller and can be highly integrated. For the injection of the solution sample, we defined the inlet and outlet, respectively. The height of the cylindrical cavity was 500 μm, and the thickness of PDMS was about 3 mm. With the constant injection of the solution sample, the output light power will change when different refractive index solutions are injected into the cavity. Because RI can be adjusted in a wide range with the change of sucrose concentration, the real-time detection of sucrose solution can be carried out through the optical power meter console (PM400, Thorlabs; Shanghai, China).

**Figure 3:**
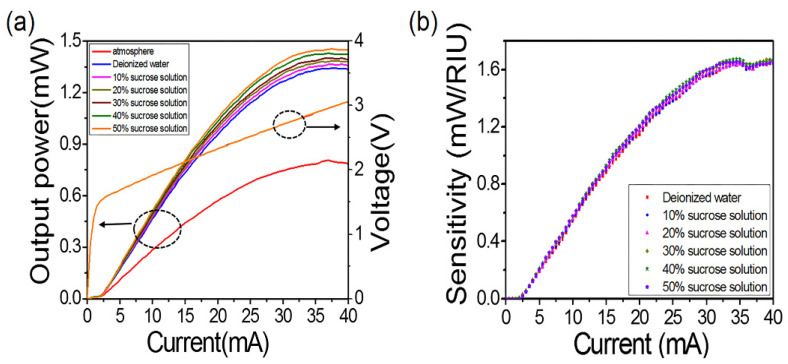
(**a**) P-I-V characteristics of sensor under different conditions. (**b**) Sensitivity curve of sensor and air environment.

Next, we characterized the performance of the refractive index sensor. It included P-I-V and a real-time power (P)—time (T) curve. According to the P-I-V curve, the threshold current was 2.5 mA, and the differential resistance was 39.4 Ω. Figure 3a shows the P-I-V curve with constant voltage and current and variable refractive solution index. It can be seen that the output light power also changed when different refractive index solutions were introduced. It shows that the sensor was responsive to the change of the background refractive index. Figure 3b shows the change in sensor sensitivity when different refractive index solutions were introduced. Here, we defined the refractive index sensitivity s as the ratio of light intensity change to volume RI change, s = ΔP/Δn. From the test results, when the saturation current was 40 mA, the sensitivity reached the maximum value of 1.65 × 10^6^ nW/RIU, which was an order of magnitude higher than the sensitivity of 1.1 × 10^5^ nW/RIU in Zhao et al.’s article. Therefore, with the optimized gold nanoparticles, the quality of the sensing layer was higher, which improved the overall sensing sensitivity of the device. As shown in Figure 3b, the sensitivity increased accordingly, when the input current increased. In addition, the overlapping curve shows the stability of the sensor. The experimental results show that when the input current was constant, the optical output power of the sensor had a linear relationship with RI.

### 3.3. Stability Test Results

**Figure 4:**
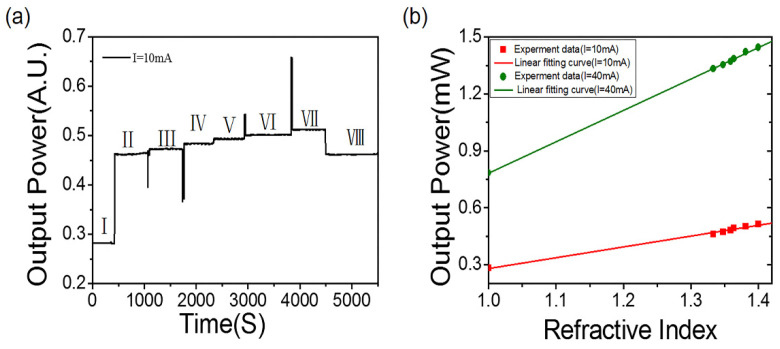
(**a**) Time response of LSPR sensor to different RI (I = 10 mA) sucrose solutions. (**b**) RI response of LSPR system under different input currents.

To verify the stability of the sensor and obtain the highest sensitivity, sucrose solutions with RIs of 1.3333, 1.3477, 1.3588, 1.3638, 1.3811 and 1.3988 were successively fed into the microfluidic system. The output light response was recorded, and the sensitivity was achieved. Figure 4a shows the light intensity response of solutions with different refractive index (RI) When the input current was 10 mA, the output power was 0.282 mW. When the input current was constant, the linear response of the optical output power to RI was between 1.3333 and 1.3998 from stage II to stage VII. In stage VIII, deionized water with RI of 1.3333 ws reintroduced. Since the two environmental refractive indexes are the same, the output optical power obtained was the same as that in stage II, indicating that the sensor devices could be reused. Figure 4b shows the sensor’s performance with the different input currents. When the input current was 40 mA, the sensitivity of S = 1.65 × 10^6^ nW/RIU reached the greatest value. The fitting coefficient (R^2^) is 0.9994, and the fitting equation is y = 1.654x − 0.871.

## 4. Conclusions

In conclusion, we proposed a novel refractive index sensor. The excitation light source (vertical-cavity surface-emitting laser), sensitive sensing layer (gold nanostructure), and microfluidic channel were integrated into one chip to develop an LSPR sensor chip with small volume, low cost, and high sensitivity. Compared with the traditional LSPR sensor, the novel refractive index sensor proposed in this work innovatively integrated the 850 nm laser with the gold nanoparticle array as the sensing layer. AAO films were applied for preparing the gold nanoparticle arrays. The sensitivity of the LSPR sensor to sucrose solution with a refractive indexes of 1.333–1.3998 was tested. The test results showed that the sensor had a certain responsivity to the background refractive index and its performance was stable and repeatable. The maximum sensitivity calculated by the light intensity change rate was 1.65 × 10^6^ nW/RIU. The experimental results were also verified by FDTD simulation. The sensor has the advantages of simple preparation, miniaturization, portability, repeatability, and high integration, which provides a new idea for the future direction of sensors and has great potential applications.

## Data Availability

Data are available in the main text.

## Supplementary Materials

The following supporting information can be downloaded at: https://www.mdpi.com/article/10.3390/nano12152607/s1, Figure S1: The preparation process of this integrated sensor. (a) SiO_2_ deposit. (b) Photolithographic etching to produce optical cell patterns. (c) Etching GaAs substrate. (d) Wet oxidation and Corrosive SiO_2_. (e) SiO_2_ deposit. (f) Open light hole. (g) Sputtering electrode; Figure S2: Simulation results of transmittance of gold nanoparticles. (a) Simulation results of transmittance of gold nanostructures with diameter of 280 nm and period of 450 nm. (b) The transmittance of gold nanostructures was tested experimentally. (c) Simulation results of transmittance of gold nanostructures.
