# Supplementary material for: An LSPR Sensor Integrated with VCSEL and Microfluidic Chip
Source: Nanomaterials (Basel). 2022 Jul 29;12(15):2607. doi: 10.3390/nano12152607 (PMC9370176; doi:10.3390/nano12152607)
Supplement: Supplementary file 1 [file nanomaterials-12-02607-s001.zip › nanomaterials-1830220-supplementary.pdf]

Supplementary Materials

An LSPR Sensor Integrated with VCSEL and Microfluidic Chip

Fang Cao [1,†], Xupeng Zhao [1,†], Xiaoqing Lv [2,*], Liangchen Hu [1], Wenhui Jiang [1], Feng Yang [1], Li Chi [3,*], Pengying Chang [1], Chen Xu [1] and Yiyang Xie [1,*]

[1] Key Laboratory of Optoelectronics Technology, Ministry of Education, Beijing University of Technology, Beijing 100124, China; caofang950318@163.com (F.C.); zhaoxp2311@163.com (X.Z.); huliangchen163@163.com (L.H.); jiangwenhui1997@163.com (W.J.); yangfengqs@bjut.edu.cn (F.Y.); pychang@bjut.edu.cn (P.C.); xuchen58@bjut.edu.cn (C.X.)

[2] State Key Laboratory of Integrated Optoelectronics, Institute of Semiconductor, Chinese Academy of Sciences, Beijing 100083, China

[3] School of Traditional Chinese Medicine, Capital Medical University, Beijing 100069, China

* Correspondence: lvxiaoqing284@semi.ac.cn (X.L.); chili@ccmu.edu.cn (L.C.); xieyiyang@bjut.edu.cn (Y.X.); Tel.: +86-10-67391641-868 (Y.X.)

† These authors contributed equally to this work.

VCSEL preparation process

The preparation process of the 850 nm band vertical cavity surface emitting laser used as the sensing light source is as follows: first, after cleaning the epitaxial wafer, deposit a layer of SiO_2 on its p surface, as shown in step (a) in the figure. After that, the circular light emitting unit pattern of VCSEL is lithographed, and then the SiO_2 mask for protecting the table surface is left through steps such as etching SiO_2 and cleaning. In the next step, the epitaxial sheet is dry etched with ICP equipment, and the etching depth is about 3.2 μm expose the active area of VCSEL. Wet oxidation is carried out after etching, and then a layer of SiO_2 is passivated on the surface of the epitaxial wafer after oxidation as an insulating layer. Next, two negative photoresist lithography processes are needed to prepare the light outlet hole and front electrode of VCSEL, and then sputter the front and back electrodes respectively. Finally, the VCSEL is annealed to improve the ohmic contact and reduce the differential resistance, so as to improve the performance of the laser.

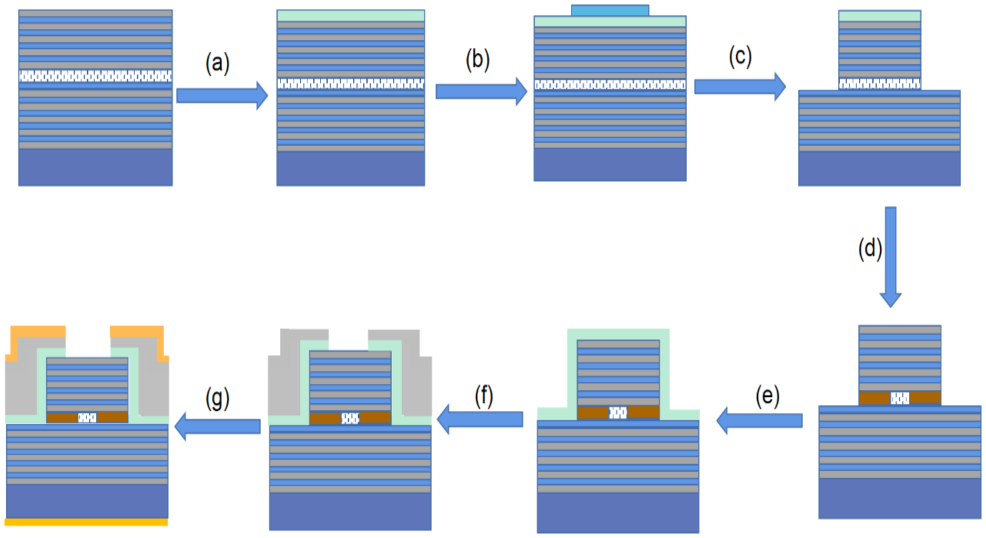

Figure S1. The preparation process of this integrated sensor. (a) SiO_2 deposit. (b) Photolithographic etching to produce optical cell patterns. (c) Etching GaAs substrate. (d) Wet oxidation and Corrosive SiO_2. (e) SiO_2 deposit. (f) Open light hole. (g) Sputtering electrode.

Firstly, the time-domain finite element difference algorithm FDTD is used to simulate and optimize the parameters of gold nanostructure, and finally the structure that best matches the 850nm laser is selected, that is, the diameter of gold particles is 280nm and the period is 450nm, as shown in Fig. 2 (a). Subsequently, gold nanostructures were prepared on the quartz sheet and tested to obtain the simulation results. As shown in Fig. 2 (b), it can be seen that there is a certain difference between the formant and the simulation results. The actually prepared structures were measured to obtain gold nanoparticles with a diameter of 400 nm and a period of 600 nm. Therefore, the FDTD simulation with this parameter obtains the results of Fig. 2 (c). Comparing Fig. 2 (b) and Fig. 2 (c), the formant positions are roughly the same, and the transmittance increases with the increase of refractive index. It shows that the simulation results can support the experimental results. It is observed that the transmittance spectral lines of the three diagrams are red-shift with the increase of refractive index.

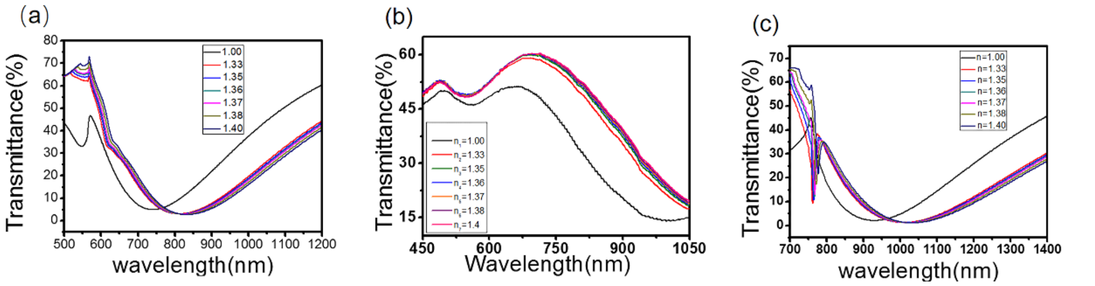

Figure S2. Simulation results of transmittance of gold nanoparticles. (a) Simulation results of transmittance of gold nanostructures with diameter of 280nm and period of 450nm. (b) The transmittance of gold nanostructures was tested experimentally. (c) Simulation results of transmittance of gold nanostructures.
